# Analysis of T and NK cells immune response in Ipilimumab treated Melanoma patients

**DOI:** 10.1186/1479-5876-13-S1-O8

**Published:** 2015-01-15

**Authors:** Rossana Tallerico, Costanza M Cristiani, Mariaelena Capone, Gabriele Madonna, Domenico Mallardo, Ester Simeone, Andrea Dominijanni, Antonio M Grimaldi, Francesco Colucci, Paolo A Ascierto, Ennio Carbone

**Affiliations:** 1 Tumorimmunology and Immunopathology Laboratory, Department of Experimental and Clinic Medicine, University "Magna Graecia" of Catanzaro, Campus - Germaneto, 88100 Catanzaro Italy; 2 Melanoma. CancerImmunotherapy and Innovative Therapy Unit, Istituto Nazionale Tumori Fondazione "G. Pascale", Via Mariano Semmola, 80131 Napoli –Italy; 3 Immunohaematological Transfusion Medicine Centre “Pugliese-Ciaccio” Hospital of Catanzaro, 88100 Ctanzaro, Italy; 4 Department of Obstetrics and Gynaecology, University of Cambridge Clinical School, Cambridge, UK; 5 Department of Microbiology Cell and Tumorbiology (MTC), Karolinska Institutet, Nobelvag 16, 17077 Stockholm, Sweden

## Background

The most promising immunotherapeutics tested in metastatic melanoma patients are the monoclonal antibody blocking CTLA-4 (Ipilimumab), and those interfering with PD-1 and PD-L1. However, the lack of knowledge on predictive biomarkers that could assist the treatment remains a limiting factor. We speculate that, along with additional markers, the immunoscore [1] is fundamental as prognostic and predictive marker for response to immunotherapies in metastatic melanoma. Our previous data demonstrate that NK cells control the melanoma progression [2,3]. Therefore we have analysed both T cells and NK cells subsets frequencies and receptors repertoire in the peripheral blood of Ipilimumab treated patients with Stage IV metastatic melanoma.

## Material and methods

Peripheral blood mononuclear cells (PBMCs) from 12 different patients with stage IV metastatic melanoma were collected and analyzed. Each patient received 4 infusions of Ipilimumab each 21 days. Before each infusion we collected patients’ blood and isolated PBMCs to analyze the lymphocytes compartment.

We stained for the following antibodies: CD56 PE, CD3 Fitc, CD56 APC, CD4 PeCy7, CD8 APCCy7, CD152 (CTLA4) PE, CD279 (PD-1) PE, CCR7 PeCy7, CD158a/h(KIR2DL1/S1) PE, CD158b (KIR2DL2/DL3) PE, CD158e (KIR3DL1) PE, CD16 APCCy7, CD57 PE, CD69 PE, CD314 (NKG2D) PE, CD226 (DNAM-1) PE, CD337 (NKp30) PE, CD336 (NKp44) PE, CD335 (NKp46) PE (Miltenyi Biotech), CD192 (CCR2) AlexaFluor 647, CXCR2 (IL8RB) APC, 7-AADStaining Solution (BD Italia), TIM3 PE (ebioscience), NKG2C PE (R&D Systems). The analysis was performed with FACS CANTO II. Statistical analysis was performed with Anova and Student’s t-test.

## Results

Our data indicate that, after the first Ipilimumab treatment, an inversion of CD4/CD8 ratio occurs with a concomitant increase in the CD56^dim^ population and a higher expression of TIM-3 and NKp46 molecules on the surface of NK cells. Moreover, the frequency of NK and T cells expressing KIRs and CCR7 is reduced, while the mean fluorescence intensity of CD16 and PD1 is upregulated on both CD56^bright^ and CD56^dim^ NK cells.

## Conclusions

These preliminary data indicate that early during Ipilimumab treatment, cytotoxic lymphocytes CD8^+^ T cells and CD56^dim^ NK cells expand and become activated. NK cells seem to be polarized towards a CD56^dim^CD16^bright^ KIRs^+^NKp46^+^TIM3^+^ phenotype. Ipilimumab treatment may induce NK cells maturation, which might in turn drive activation of CD8^+^ T cells.
